# Feasibility and acceptability of virtual reality for cancer pain in people receiving palliative care: a randomised cross-over study

**DOI:** 10.1007/s00520-022-06824-x

**Published:** 2022-01-21

**Authors:** Philip D. Austin, Philip J. Siddall, Melanie R. Lovell

**Affiliations:** 1 Department of Palliative Care, HammondCare, Greenwich Hospital, Sydney, New South Wales Australia; 2 Department of Pain Management, HammondCare, Greenwich Hospital, Sydney, New South Wales Australia; 3 Sydney Medical School-Northern, University of Sydney, Sydney, New South Wales Australia

**Keywords:** Virtual reality, Cancer pain, Palliative care, Pain relief, Presence

## Abstract

**Context:**

Pain management in palliative care remains inadequate; the development of innovative therapeutic options is needed.

**Objectives:**

To determine the feasibility and preliminary effectiveness for larger randomised controlled trials of 3D head-mounted (HMD) virtual reality (VR) for managing cancer pain (CP) in adults.

**Methods:**

Thirteen people receiving palliative care participated in a single-session randomised cross-over trial, after which they completed a qualitative semi-structured interview. We also compared the effects of 3D HMD VR and 2D screen applications on CP intensity and levels of perceived presence. Feasibility was assessed with recruitment, completion rates and time required to recruit target sample.

**Results:**

Although recruitment was slow, completion rate was high (93%). Participants reported that the intervention was acceptable and caused few side effects. Although participants reported significantly reduced CP intensity after 3D HMD VR (1.9 ± 1.8, *P* = .003) and 2D screen applications (1.5 ± 1.6, *P* = .007), no significant differences were found between interventions (−.38 ± 1.2, 95% CI: −1.1–.29, *P* = .23). Participants reported significantly higher levels of presence with the 3D HMD VR compared to 2D screen (60.7 ± SD 12.4 versus 34.3 ± SD 17.1, mean 95% CI: 16.4–40.7, *P* = .001). Increased presence was associated with significantly lower pain intensity (mean 95% CI: −.04–−0.01, *P* = 0.02).

**Conclusions:**

Our preliminary findings support growing evidence that both 3D and 2D virtual applications provide pain relief for people receiving palliative care. Given the relative lack of cybersickness and increasing access to portable VR, we suggest that larger clinical studies are warranted.

**Supplementary Information:**

The online version contains supplementary material available at 10.1007/s00520-022-06824-x.

## Introduction

Pain is the most common reason for out-of-hours care and hospitalisation in people with advanced cancer [1]. Recent meta-analysis shows pain prevalence in people with cancer at 66% in advanced metastatic disease compared to 39% after curative treatment and 55% during anticancer treatment [2]. While patients prefer care at home, data unfortunately show that here is where pain control is poorest and is undertreated in about one-third of patients [3]. Pharmacological treatments for CP show significant adverse effects on quality of life plus the potential interactions with anti-cancer drugs [4, 5]. Psychological factors further contribute to the experience of CP where distress is shown to significantly increase pain severity and pain-related disability [6]. Despite best practice advocacy for pain management in palliative care settings [7], there remains a significant burden of unrelieved pain. Thus, development of innovative alternative therapeutic options is needed.

One option gathering interest is virtual reality (VR). VR is a computer-simulated creation of a 3D environment [8] and is an effective treatment for medical and psychological conditions such as post-stroke rehabilitation and anxiety [9, 10]. Current VR systems include head-mounted devices (HMDs) with 3D-enabled glasses with additional devices including headphones for noise-cancelling and sound, head and/or body-tracking sensors and other input hardware such as joysticks and data gloves [11]. Together these form a realistic multisensory experience described as a real or simulated environment where the perceiver experiences a sense of presence, defined as an illusion of ‘being there’ [12]. Investigations into the mechanisms of VR analgesia in experimental settings show that the degree of analgesic effect is dependent on the user’s sense of presence in the virtual environment [13]. Moreover, meta-analysis shows no difference in effectiveness between specifically developed software and commercially available games using 3D environments [14], thus increasing access to affordable VR options for use in experimental and clinical pain settings.

Increasing evidence shows VR to be an effective analgesic intervention for chronic pain conditions including fibromyalgia and spinal cord injury pain [15] and acute pain situations, including labour and procedural pain [16]. VR is also effective in reducing often frustrating comorbid symptoms including anxiety and reduced self-efficacy [17]. Thus, it has been proposed that VR may be an effective and relatively harmless adjunct for the management of pain in people with cancer and works via distraction [18].

Currently, only two studies using VR applications show decreases in pain in cancer populations. First, Mohammad and colleagues show VR as an adjunct intervention is more effective than morphine alone in relieving pain and anxiety in women with breast cancer [19]. Second, Niki and colleagues found that VR travel was effective in improving pain and other cancer-related symptoms in people with terminal cancer.

No randomised studies comparing 3D HMD and 2D screen applications in people with CP exist. Evidence suggests that 3D VR technologies are more realistic than 2D where 3D perception of a VR scene give people a greater sense of presence [20]. We aim to evaluate the feasibility of (a) recruitment for VR and data collection procedures in people receiving palliative care both in hospital and at home, (b) acceptability and comfort of VR devices, (c) completion rates of virtual interventions and (d) provide information for future work in terms of estimating sample size. We also aimed to determine whether (a) using a 3D HMD VR device results in a significant reduction in CP compared to a 2D screen-based device running the same VR application and (b) determine whether the level of presence in the virtual environment predicts the degree of analgesic effect in people with CP. We hypothesised 3D HMD VR would result in significantly greater analgesia compared to a 2D screen-based application and that this is associated with greater levels of presence.

## Methods

### Study design

Using a within-subject, randomised cross-over feasibility trial, two sequential interventions were compared, one with 3D HMD VR and one with 2D screen applications using the same virtual environment in a convenience sample of 14 people with CP. Research staff (PA) generated random allocation for the first administered intervention using sequentially numbered, opaque sealed envelopes. Due to differences in appearance between the two interventions, participant and researcher blinding were not possible; however, because it was important for researchers to show parity in describing both interventions, a script using neutral language was prepared.

This study was an investigator-initiated feasibility trial funded by the Sydney Vital reference 201142 and was registered by the Australia New Zealand Clinical Trials Registry, number ACTRN 12619000568112p in April 2019. The Northern Sydney Local Health District Research Ethics Committee approved this single-site feasibility trial in March 2020, reference 2019/ETH12454. Study reporting complies with CONSORT guidelines [21].

- Feasibility was assessed by collecting data on the rate of completion across both interventions. Feasibility was defined as 80% completion [22]. Feasibility of data collection methods was assessed by determining completion rates of trial outcome collection again defined as 80% completion.
- Acceptability of VR devices was determined using a semi-structured interview.

### Semi-structured interview

Participants were individually interviewed at study completion using an semi-structured interview guide adapted from Pedroli and colleagues [23] to explore participant experiences and challenges using the VR platforms (Table 1). The interview included four topics: VR usability, presence, cybersickness and expectations. For the usability topic, sub-themes included questions on user application, ease and challenges of learning and satisfaction. Concerning the presence topic, sub-themes included questions on spatial awareness, user engagement and VR realism. Cybersickness and expectations questions related to symptoms felt during and after each application, expected amount of VR use in the future and finally, the use of VR for any other symptoms or complaints.

**Table 1:** Topics, sub-topics and questions of semi-structured interview (adapted from Pedroli et al 2018)

Topic	Subtopic	Questions
Usability	Application	1. How did you find using the hand-held device? 2. How did you find moving around the virtual environment? 3. Were there any technical problems with the headset and/or hand-held devices during the VR session?
	Learning	4. Did you ask or need help to understand how to use the VR headset and/or hand-held devices during the VR session?
	Satisfaction	5. What did you like about the virtual environment? 6. How did you feel during the VR session?
Presence	Spatial awareness	7. How immersed did you feel in the virtual environment? 8. How much control did you have when moving and observing the virtual environment?
	Engagement	9. What did you think about during the VR session? 10. Did you get distracted by noises outside the virtual environment?
	Realism	11. How did you find the virtual environment, realistic or too artificial?
Cyber sickness	Physical effects	12. Did you feel ill during the VR session? 13. If yes, did you have nausea, dizziness or other physical symptoms during the VR session?
Expectations	Future use	14. How often would you use virtual reality for pain relief in the future?
	Other uses	15. Would you use virtual reality for any other complaints such as negative feelings and/or physical symptoms?

### Participants

Eligible participants were

- Palliative care inpatient unit patients or patients receiving home-based palliative care,
- Aged 18 years and older,
- A diagnosis of cancer,
- The presence of cancer-related pain over the previous 24 hours and at the time of study participation,
- Life expectancy of one month and over.

Exclusion criteria included

- The presence of other types of pain more prominent at the time of study,
- Psychiatric comorbidities not including anxiety, depression or stress,
- Legally blind,
- History of motion sickness.

Participants were identified by hospital and community clinical staff, whereupon they were given participation information. Upon signed consent, participants completed a prevalidated case report form. Additionally, ongoing CP management was not altered prior to or during the study period. Five people meeting eligibility criteria declined to participate due to exacerbation in breakthrough symptoms (n-3) and unwillingness of loved ones for patient participation (n-2).

### Study schedule

**Figure 1:**
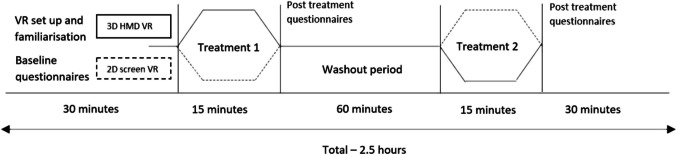
Cross-over study protocols for 3D HMD and 2D screen VR intervensions. A cross -over timeline diagram of the VR protocols that includes VR set-up and baseline questionnaire completion, first VR session, between-VR application washout period, second VR session and post-VR session questionnaire completion

**Figure 2:**
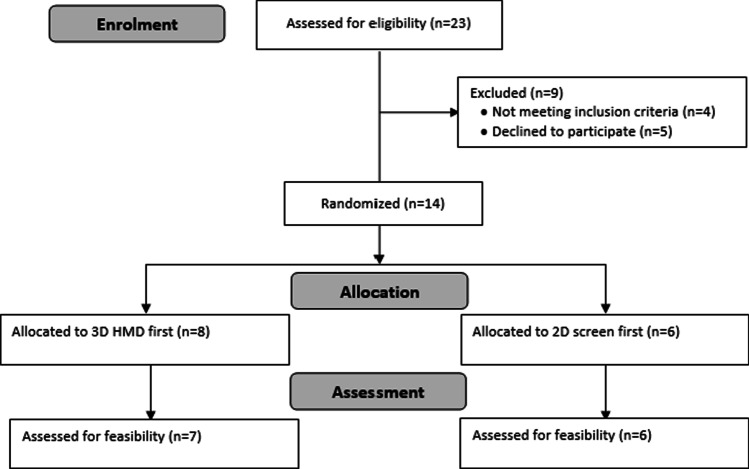
CONSORT flow diagram showing participant enrolment, allocation and assessment for feasibility

Baseline CP measures were taken for average, worst and least CP intensities over the previous 24 hours, in addition to current CP intensity. Importantly, participants were instructed only to rate their CP. We compared differences in effect between 3D HMD and 2D screen devices using the same VR software on CP where the duration for each intervention was 15 min. The primary CP score was reported immediately after each intervention while additional pain data was collected at 5, 10 and 20 min after each intervention. Lowest and average CP scores during interventions were also reported. To eliminate any carryover effect from the first intervention, a washout period separated the two treatment periods (see Figures 1, 2). Here, participants were free to move around their surroundings. Because washout periods need to be at least five times the half-life of a given treatment [24], we chose 60 min, based on data showing that pain is significantly reduced immediately after VR exposures but lasts no longer than 10 min after VR exposure [25]. Participants were required to report headset discomfort and cyber-sickness (includes symptoms of nausea, vomiting, headache and vertigo) prior to, during or after using both applications.

### VR interventions

#### 3D HMD VR application

The Oculus Rift® headset is commercially available and widely utilised for VR studies in medical research [26]. For this study, participants viewed a 3D VR experience called Nature Trek®. Prior to use, participants were instructed by the researcher (PA) on the use of a hand-held joystick, how to move around an alpine meadow environment and make full use of the 360° scene. To avoid cybersickness, this audio-visual experience was non-interactive [27]. The application was standardised across the group. The VR headset was calibrated for participants’ eyesight in addition to advice on motion sickness prevention during VR such as reducing speed of their character and/or reducing head movement (Fig. 1).

#### 2D screen application

The same application including audio inputs was run on a 17.3-inch Alienware® laptop screen with the participant seated in the same position. This allowed for a reliable comparison between the effects of 3D VR and 2D screen experiences.

### Primary outcome measures

#### Pain numerical rating scale (NRS)

Participants completed an 11-point pain NRS after each intervention where participants not only reported levels of pain intensity after each intervention, but additionally reported their average and lowest pain intensity during each intervention. The 11-point pain NRS is a reliable and valid measure where meta-analysis shows extensive use across pain populations [28].

### Secondary outcome measures

#### Edmonton Symptom Assessment System (ESAS)

The ESAS was administered at baseline and then immediately after each VR session. The ESAS is a validated self-reporting tool used in cancer populations consisting of nine visual analogue scales measuring pain, activity, nausea, depression, anxiety, drowsiness, lack of appetite, well-being and shortness of breath in cancer and palliative care settings [29].

#### Australian-modified Karnofsky Performance Status (AKPS)

For overall performance status at baseline, we completed the AKPS at baseline only. This is a validated measure of a patient’s overall performance status using a single score between 10 and 100 [30].

#### iGroup Presence Questionnaire (IPQ)

To investigate levels of presence during each VR session, participants completed the IPQ after each application. The IPQ is a valid and reliable seven-point Likert scale for measuring spatial awareness, levels of involvement and experienced realism in a range of virtual environments ranging from 0 (Not at all) to 6 (Very much) [31].

### Data analysis

Data analyses were performed using SPSS Statistics 24 [32]. Descriptive statistics were drawn from demographic and clinical features of the sample including age, years since cancer diagnosis, performance status, cancer stage, duration of CP and type of cancer. We used linear mixed model analysis for repeated measures with post 3D HMD VR and 2D screen pain and presence scores as dependent variables for post 3D HMD VR and 2D screen time points. These regressions included a factor for the condition (3D HMD VR and 2D screen), the sequence (randomised sequence of conditions between subjects) and time (randomised sequence of conditions within subjects), where sequence was modelled as a random effect. The analysis controlled for baseline (pre-randomisation) pain intensity in models where post-intervention pain intensity was the dependent variable [33]. We also used paired sample T-test to compare pre-post mean difference scores for pain, presence and ESAS items within each treatment condition. Thematic analysis was used to identify, organise, describe and report themes arising from the semi-structured interview [34].

## Results

Between July 8, 2020, and May 13, 2021, only eight patients in community care and 15 in hospital care were contacted to participate in this study of whom 14 were enrolled in the study (Table 1). The main reason for such slow recruitment was the presence of the COVID-19 pandemic. One participant withdrew during the study due to excess levels of non-cancer-related low back pain when sitting at the first intervention, giving a 93% study completion rate. Table 2 shows participants’ demographic characteristics. Baseline, post-intervention, mean differences and p-values for 3D HMD VR and 2D screen for CP intensity and ESAS items are shown in Table 3. Figure 2 shows recruitment and dropout of participants during the study.

**Table 2:** Baseline characteristics of 14 participants with cancer pain receiving palliative care.

Baseline Characteristics	Mean (SD)
Age	71.1 (14.3)
Years with cancer	6.0 (4.2)
Months with cancer pain	15.8 (9.4)
AKPS score	66.9 (14.9)
Cancer pain levels over previous 24 h (0–10)	
• Average	4.8 (2.2)
• Worst	6.1 (2.5)
• Least	2.7 (2.1)
	**Number**
Gender	
• Female	6
• Male	8
Patient-type	
• Inpatient	11
• Outpatient	3
Primary cancer	
• ENT	2
• Neuroendocrine	1
• Pancreatic	2
• Colorectal	2
• Breast	1
• Prostate	2
• Cervical	1
• AML	1
• Liposarcoma	1
• Ovarian	1
Stage of illness	
• Stage 4	14
Cancer therapy (past and current)	
• Chemotherapy	2
• Radiotherapy	4
• Combined	8
Type of care	
• Symptom management	11
• End of life care	3
Cancer pain location	
• Face/throat	2
• Abdomen	2
• Spine/ribs	3
• Pelvis	6
• Hips	1
Cancer pain consistency	
• Constant	8
• Intermittent	6

Abbreviation – Australian-modified Karnofsky Performance Status (AKPS)

**Table 3:** Baseline and post 3D HMD VR and 2D computer screen mean scores, mean differences, confidence intervals and *p*-values for palliative care symptoms using paired T-Test analysis

Mean (SD)			3D Head mounted VR				2D computer screen			
	Baseline	During and post-treatment	Mean (SD)	Mean diff (SD)	95% CI of difference	P-value	Mean (SD)	Mean diff (SD)	95% CI of difference	P-value
Cancer pain intensity	3.6 (1.4)	**Average pain during**	1.6 (1.6)	2.0 (1.7)	.98–3.1	.001	1.9 (1.7)	1.7 (1.4)	.87–2.5	.001
		**Least pain during**	.9 (1.5)	2.7 (1.8)	1.6–3.8	.0002	1.2 (1.8)	2.5 (1.6)	1.5–3.4	.0001
		**Immediately post**	1.8 (1.5)	1.9 (1.8)	2.9–3.8	.003	2.2 (1.8)	1.5 (1.6)	2.4–3.3	.007
		**5 min post**	2.3 (1.5)	1.3 (1.8)	.19–2.4	.025	1.9 (1.9)	1.8 (1.9)	2.3–3.3	.005
		**10 min post**	2.5 (1.7)	1.2 (2.2)	−.17–2.5	.082	2.1 (1.7)	1.5 (1.3)	.74–2.3	.001
		**20 min post**	2.2 (1.8)	1.4 (1.7)	.38–2.4	.011	2.4 (1.8)	1.2 (1.4)	.37–2.1	.009
Tiredness	6.1 (2.3)		2.2 (2.8)	3.8 (3.9)	1.5–6.1	.004	3.2 (3.1)	2.8 (3.9)	.47–5.2	.023
Drowsiness	5.4 (2.8)		1.5 (2.2)	3.9 (3.3)	1.9–5.9	.001	2.8 (2.9)	2.6 (3.8)	.35–4.9	.027
Nausea	1.5 (2.5)		1.9 (2.9)	−.39 (3.3)	−2.1–1.4	.64	1.0 (2.2)	0.5 (1.8)	−.55–1.6	.30
Lack of appetite	4.4 (3.9)		4.9 (4.2)	−.53 (1.2)	−1.3−.23	.15	4.2 (4.1)	0.2 (2.0)	−1.1–1.4.	.79
Shortness of breath	2.8 (2.4)		.77 (1.1)	2.0 (2.3)	.63–3.4	.008	1.1 (2.0)	1.7 (1.7)	.66–2.7	.004
Depression	2.2 (2.7)		0 (0.0)	2.2 (2.7)	.52–3.8	.014	0.2 (0.4)	.19 (2.6)	.35–3.5	.02
Anxiety	1.9 (2.5)		0.2 (0.6)	1.8 (2.6)	.23–3.3	.028	0.4 (0.7)	1.5 (2.1)	.27–2.8	.02
Wellbeing	3.4 (1.9)		1.2 (1.8)	2.2 (2.5)	.66–3.7	.009	1.8 (1.6)	1.6 (2.1)	.34–2.9	.02

### Semi-structured interview

Twelve of 13 participants completing the study answered open-ended questions about their experiences using both platforms. One participant reported fatigue after the study and was unable to complete the interview. Although participants reported initial difficulties using the hand-held device, they could move around the VR environment easily after a few minutes practice. Two participants required instruction over the duration of the study. All participants described satisfaction related to pleasure, peacefulness, enjoyment, escape, relaxation and most positively, childhood memories, especially using 3D HMD VR. All participants reported high levels of immersion with most reporting competent control enabling them to see as much virtual nature as possible. Only two participants were aware of external noise distractions. Although three participants reported virtual features including flora and sky as two-dimensional, all described the virtual environment as being ‘very realistic’. Two participants reported mild non-limiting nausea with 3D HMD VR, while a further two reported initial but transient dizziness in the virtual environment. Finally, most participants stated they would use VR regularly if available for pain but also for emotional relief and relaxation.

### Effects of 3D HMD VR and 2D screen applications on cancer pain intensity

Paired sample T-test analysis showed 3D HMD VR and 2D computer screen applications separately produced significant reductions in CP intensity immediately after both interventions (1.9 ± 1.8, *P* = .003 and 1.5 ± 1.6, *P* = .007). Participants additionally reported pain relief at 5, 10 and 20 min relative to baseline after the 2D computer screen (*P* = .005, *P* = .001 and *P* = .009 respectively) compared to pain relief with 3D HMD VR relative to baseline at the same time points (*P* = .02, *P* = .08 and *P* = .01 respectively) (Table 3). At post-intervention, participants reported a mean decrease in pain of 43% using 3D HMD VR compared to 34% using the 2D screen application. Linear mixed model analysis showed no significant difference between 3D HMD VR and 2D screen applications in reductions in CP intensity either immediately after (−0.38 ± 1.2, 95% CI: −1.1–0.29, *P* = .23), or at 5-, 10- and 20-min post-intervention (Table 3). Although the effects of randomised VR condition sequencing both between and within-subjects on pain scores were not significant immediately after (*P*=.07 and *P*=.43 respectively), at 10- and 20-minutes post-interventions, between-subject sequencing effects were significant at five minutes post-intervention (*P* = .04) (Table 4).

**Table 4:** A table showing linear mixed model analysis for the effects of VR conditions on (a) reported cancer pain intensity immediately, and at 5, 10 and 20 min, (b) reported levels of presence, (c) effects of reported levels of presence on post VR condition neuropathic pain intensity and (d) as above but showing adjustment for VR condition

Parameter	95% CI (covariate-adjusted)		*t*	*df*	Significance
	Lower bound	Upper bound			
Effect of VR conditions on immediate post VR reported pain intensity (fixed factors – condition, sequence, time)					
• 3D HMD VR vs 2D screen application	−1.10	.28	−1.2	13	.23
• Sequence (between subject)	−3.10	.14	−2.0	12	0.7
• Time (within subject)	−.43	.95	.82	12	.43
Effect of VR conditions on 5 min post VR reported pain intensity (fixed factors – condition, sequence, time)					
• 3D HMD VR vs 2D screen application	−1.2	.28	−1.4	12	.20
• Sequence (between subject)	−3.0	−.08	−2.2	20	.04*
• Time (within subject)	−.71	.78	.11	12	.92
Effect of VR conditions on 10 minutes post VR reported pain intensity (fixed factors – condition, sequence, time)					
• 3D HMD VR vs 2D screen application	−.59	1.2	.81	11	.43
• Sequence (between subject)	−3.1	.01	−2.2	11	.06
• Time (within subject)	−.42	1.4	1.2	11	.25
Effect of VR conditions on 20 minutes post VR reported pain intensity (fixed factors – condition, sequence, time)					
• 3D HMD VR vs 2D screen application	−.64	.36	−.62	11	.54
• Sequence (between subject)	−3.0	1.2	−.91	11	.38
• Time (within subject)	−.65	.36	−.63	11	.54
Effect of VR conditions on post VR reported levels of presence (fixed factors – condition, sequence, time)					
• 3D HMD VR vs 2D screen application	14.3	38.2	4.8	11	.001*
• Sequence (between subject)	−8.7	33.2	1.4	7	0.21
• Time (within subject)	−10.2	13.7	.32	11	.75
Effect of reported levels of presence on post VR reported pain intensity (fixed factors – presence, sequence, time)					
• IPQ score	−.04	−0.01	−2.7	12	.02*
• Sequence (between subject)	−2.8	.37	−1.7	11	.12
• Time	−.87	.65	−0.3	14.6	0.76
Effects of VR conditions on reported levels of presence on post VR reported pain intensity (adjusted for VR condition)					
• 3D HMD VR vs 2D screen application	−.42	1.64	1.3	13	.22
• IPQ score	−.07	−.01	−2.7	14	.02*
• Sequence (between subject)	−.2.6	.52	−1.4	12	.17
• Time (within subject)	−.30	.96	1.2	10	.28

### Effects of 3D HMD VR and 2D screen application on levels of presence

Linear mixed model analysis showed that 3D HMD VR was associated with significantly greater levels of presence compared to a 2D screen application (60.7 ± SD 12.4 versus 34.3 ± SD 17.1, mean 95% CI: 16.4–40.7, *P* = .001) (Supplementary Figure 2). Furthermore, the same analysis showed that increases in levels of reported presence were associated with greater reductions in pain intensity, regardless of randomised sequencing of VR conditions (*P* = .02). Additionally, even when VR condition and presence are controlled for, the effect of presence on changes in pain intensity remains significant (*P* = .02) (Table 4).

### Effects of 3D HMD and 2D screen applications on general symptoms found in palliative care

Paired T-test analysis showed that 3D HMD VR and 2D screen applications separately produced significant reductions in some symptoms such as drowsiness (3.8 ± 3.3, *P* = .001 versus 2.6 ± 3.8, *P* = .03) and shortness of breath (2.0 ± 2.3, *P* = .008 versus 1.7 ± 1.7, *P* = .004). 3D and 2D applications also showed significant improvements in wellbeing (2.2 ± 2.5, *P* = .009 versus 1.6 ± 2.1, *P* = .02) (Table 3). Conversely, no significant differences were shown for 3D and 2D applications for other symptoms such as nausea (*P* = .64 versus *P* = .30), and lack of appetite (*P* = .20 versus *P* = .80). Like the effects on CP intensity, linear mixed model analysis showed no significant differences in effects between 3D HMD VR and 2D screen applications on all ESAS symptom items.

## Discussion

This study shows feasibility and acceptability of VR for people receiving palliative care. Both 3D AND 2D platforms were well tolerated by participants who described positive experiences during and after sessions. Encouragingly, all participants reported they would use VR regularly if given the chance. Thus, given this positive feedback in association with the high completion rates, user comfort, increasing ease of access to affordable VR platforms and encouraging quantitative findings, prospect of future studies is promising. Although this feasibility study was not powered to detect differences between intervention and control, our findings indicate that participants with CP report significant decreases in CP intensity during, immediately post-intervention and up to 20 min after both interventions. However, although participants reporting significantly greater levels of presence during 3D HMD VR compared to a 2D screen session, there were no significant differences in reductions of post-treatment pain intensity between the two interventions. This may be due to participants being able to sit comfortably close to the screen using the bedside table and not feel confined by the headset.

Qualitative feedback contributes a deeper evaluation of positive and negative experiences that may explain these findings. First, high levels of user satisfaction engagement and immersion especially using 3D HMD VR support differences in levels of presence between platforms. Second, given the low level of CP intensity at baseline, participants may have had difficulty perceiving changes after each intervention. These latter findings are supported by previous work showing more moderate to severe pain at baseline predicts more significant responses to analgesic interventions [35, 36].

Findings in this study differ to previous work using the same trial applications and protocols in people with spinal cord injury pain (SCI). Here, 3D HMD VR had significantly greater analgesic effect on SCI pain compared to 2D screen applications (*P* < .0001) where participants showed over 65% decreases in pain intensity using 3D VR compared to 43% in this study [13]. The difference in sample-type may account for this variance. Compared to the long-term stability of SCI and control of neuropathic pain control, our CP sample were at varying stages of advanced disease and titrating doses of analgesia prior to the study. Our current sample were older (71.1 ± 14.3 versus 54.3 ± 14.1), frailer and reported fluctuating levels of symptom severity and pharmacological symptom control. It should also be noted that the younger SCI sample were more familiar with computer technology and thus were able to familiarise themselves with devices more quickly.

Although participants reported reductions in general symptoms, no significant differences were shown for all ESAS item scores when comparing the effects 3D and 2D interventions. Our findings for 3D HMD VR are comparable to a previous study using similar technology on people reporting CP. Using ESAS, Niki and colleagues investigated the effects of 3D HMD VR (Google Earth®) to reduce symptom severity in 20 terminal cancer patients [37]. Here, both studies report comparably significant reductions in all symptoms except nausea and lack of appetite using 3D HMD VR. Given the association between a lack of appetite and nausea in people with advanced cancer and the potential for cybersickness when using VR, these results are understandable.

In the second study exploring the analgesic effects VR on CP, Mohammad and Ahmed examined the effects of 3D HMD VR (Ocean Rift® or sitting on a beach with a ‘Happy Place’ track) plus morphine at peak effect compared to morphine alone on women with breast cancer [19]. Not surprisingly, they found significant decreases in pain intensity due to the combined analgesic effects of immersive VR and morphine. The greater effects of VR in combination with another form of analgesia are further supported by previous studies where decreases in SCI pain intensity were greater using 3D HMD VR plus transcranial direct current stimulation compared to VR alone [38, 39].

The duration of VR analgesia is an important factor. Analgesic actions of VR are divided into two types: distraction and neuroplasticity. Distraction as investigated in this study represents short-term diversion of attention away from pain towards an alternative stimulus described as ‘hijacking’ attention, emotion and memory. These effects have most recently been shown in mood-related populations where using functional magnetic resonance imaging, VR interventions decrease activity in the thalamus and limbic areas associated with anxiety and pain both acute and chronic [40, 41]. Alternatively, neuroplasticity relates to long-term functional and structural changes in neuronal pathways that occur following long-term practice of skills, such as playing a musical instrument or VR use involving interactive real-time simulations of skills or activities. In this study, we investigated short-term analgesic effects that were assessed during, immediately and up to 20 min following each intervention session. Attention distraction is most likely the best explanation for the analgesic effect of VR for pain in this study, especially concerning short-term or single session VR use.

## Limitations

First, given that this was a single-session study, and that CP is categorised as a chronic condition, it limits our ability to make strong conclusions about treatment acceptability with repeated use. Second, due to COVID-19, study recruitment was slower than anticipated. Encouragingly however, another common reason for non-participation was the successful management of pain on the ward where potential participants reported no pain at the time of assessment for eligibility. However, although the low sample size may have contributed to reduced significance in analgesic effect between the two groups, the size of the effect following 3D VR (43%) is encouraging and suggests a strong albeit temporary analgesic effect. The significant link between immersiveness and analgesia combined with the findings indicating the significantly greater sense of presence with 3D VR also supports the potential of 3D VR to produce analgesia in this setting.

Recruitment was also confounded by the instability of symptoms with advanced disease. Here a study time was arranged only for the person to withdraw on the day of the study due to exacerbation of breakthrough symptoms and the subsequent effects of pharmacology. Not surprisingly, this was a common occurrence with people nearing the end of life where only three participated in the trial compared to 10 receiving symptom management. Thus, future studies may consider sampling people receiving symptom management for investigation of more long-term analgesic effects of VR on CP.

Factors likely to have influenced our findings were the effects of advanced disease and pharmacological treatments on symptoms prior to and during the cross-over trial. Importantly, all participants engaged well with both interventions, however, there were significant differences in levels of concentration and fatigue affecting the completion of self-report measures. Although we only included three short symptom-based questionnaire, participants had difficulty recounting and/or defining symptom severity after both interventions.

## Conclusions

Our preliminary findings indicate that using VR in palliative care settings is acceptable and feasible although not without challenges. The findings also support growing evidence that virtual reality applications provide pain relief for people receiving palliative care although in the current study there was no advantage in using 3D VR when compared to 2D VR. Given the relative lack of cybersickness, the positive effects on pain and other symptoms and increasing access to portable VR, we suggest that larger clinical studies investigating more long-term use of VR in CP populations are warranted.

## Supplementary Information

ESM 1 (DOC 227 kb)

Supplementary Fig. 1 (PNG 341 kb)

High resolution image (TIFF 58 kb)

Supplementary Fig. 2 (PNG 49 kb)

High resolution image (TIFF 25 kb)

## Data Availability

All data, materials and VR hardware and software comply with field standards
