# Supplementary figures and images for: Feasibility and acceptability of virtual reality for cancer pain in people receiving palliative care: a randomised cross-over study
Source: Support Care Cancer. 2022 Jan 21;30(5):3995–4005. doi: 10.1007/s00520-022-06824-x (PMC8782583; doi:10.1007/s00520-022-06824-x)

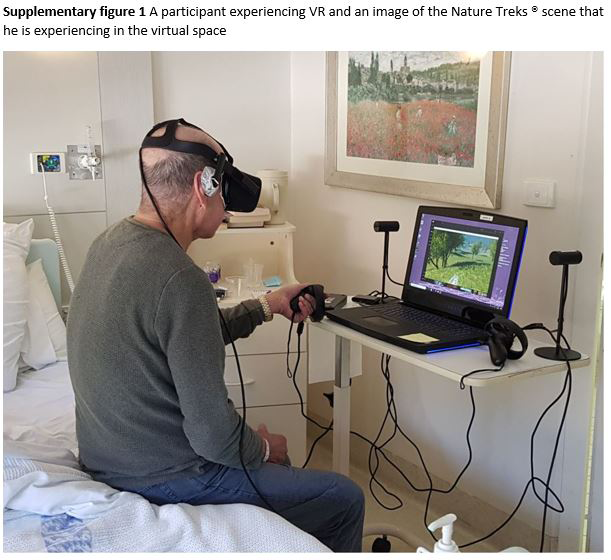

Supplement: Supplementary file 2 — (PNG 341 kb) [file 520_2022_6824_Fig3_ESM.png]

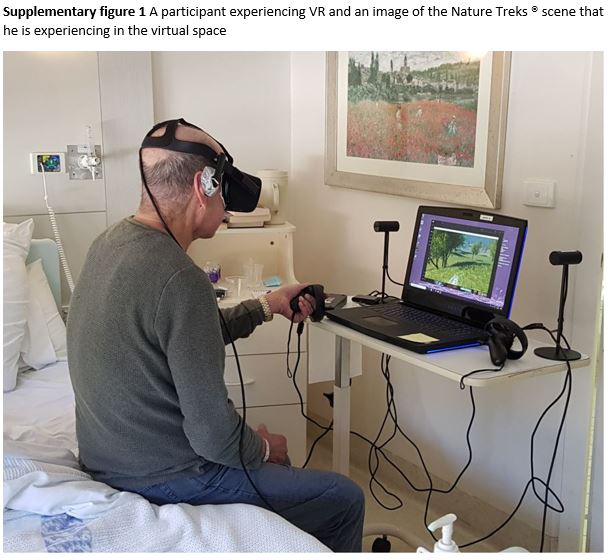

Supplement: Supplementary file 3 — High resolution image (TIFF 58 kb) [file 520_2022_6824_MOESM2_ESM.tiff]

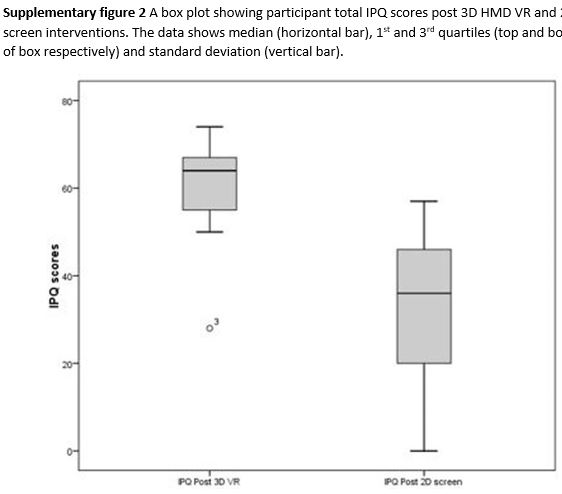

Supplement: Supplementary file 4 — (PNG 49 kb) [file 520_2022_6824_Fig4_ESM.png]

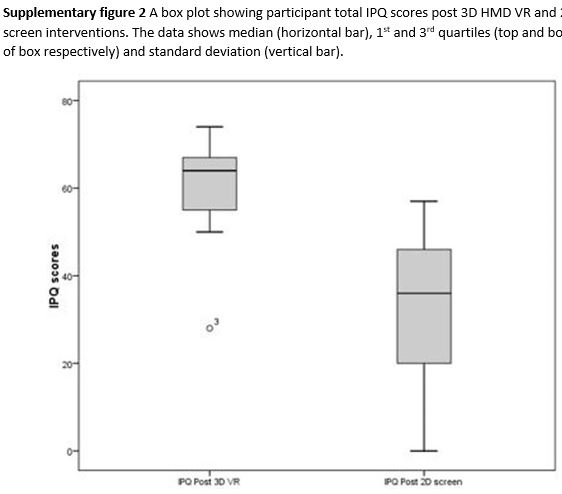

Supplement: Supplementary file 5 — High resolution image (TIFF 25 kb) [file 520_2022_6824_MOESM3_ESM.tiff]
